# Epigenetic Heterogeneity in Friedreich Ataxia Underlies Variable *FXN* Reactivation

**DOI:** 10.3389/fnins.2021.752921

**Published:** 2021-11-25

**Authors:** Layne N. Rodden, Kaitlyn M. Gilliam, Christina Lam, David R. Lynch, Sanjay I. Bidichandani

**Affiliations:** ^1^Department of Pediatrics, The University of Oklahoma Health Sciences Center, Oklahoma City, OK, United States; ^2^Oklahoma Center for Neuroscience, The University of Oklahoma Health Sciences Center, Oklahoma City, OK, United States; ^3^Division of Neurology, The Children’s Hospital of Philadelphia, Philadelphia, PA, United States; ^4^Department of Biochemistry and Molecular Biology, The University of Oklahoma Health Sciences Center, Oklahoma City, OK, United States

**Keywords:** Friedreich ataxia (FRDA), DNA methylation, *FXN* gene, histone deacetylase inhibitors (HDACi), somatic heterogeneity, epialleles, epigenetics

## Abstract

Friedreich ataxia (FRDA) is typically caused by homozygosity for an expanded GAA triplet-repeat in intron 1 of the *FXN* gene. The expanded repeat induces repressive histone changes and DNA hypermethylation, which result in epigenetic silencing and *FXN* transcriptional deficiency. A class I histone deacetylase inhibitor (HDACi-109) reactivates the silenced *FXN* gene, although with considerable inter-individual variability, which remains etiologically unexplained. Because HDAC inhibitors work by reversing epigenetic silencing, we reasoned that epigenetic heterogeneity among patients may help to explain this inter-individual variability. As a surrogate measure for epigenetic heterogeneity, a highly quantitative measurement of DNA hypermethylation via bisulfite deep sequencing, with single molecule resolution, was used to assess the prevalence of unmethylated, partially methylated, and fully methylated somatic *FXN* molecules in PBMCs from a prospective cohort of 50 FRDA patients. Treatment of the same PBMCs from this cohort with HDACi-109 significantly increased *FXN* transcript to levels seen in asymptomatic heterozygous carriers, albeit with the expected inter-individual variability. Response to HDACi-109 correlated significantly with the prevalence of unmethylated and partially methylated *FXN* molecules, supporting the model that *FXN* reactivation involves a proportion of genes that are amenable to correction in non-dividing somatic cells, and that heavily methylated *FXN* molecules are relatively resistant to reactivation. *FXN* reactivation is a promising therapeutic strategy in FRDA, and inter-individual variability is explained, at least in part, by somatic epigenetic heterogeneity.

## Introduction

Friedreich ataxia (FRDA) is characterized by progressive ataxia, cardiomyopathy, and premature mortality (Bidichandani and Delatycki, 1993). Most patients are homozygous for an expanded GAA triplet-repeat in intron 1 of the *FXN* gene (Campuzano et al., 1996). Compared to <30 GAA triplets in non-FRDA alleles, FRDA patients have 100–1500 triplets. The expanded repeat induces epigenetic silencing of the *FXN* gene (Herman et al., 2006; Greene et al., 2007; Al-Mahdawi et al., 2008), which leads to deficiency of *FXN* transcript and frataxin protein. Patients typically have 5–20% of *FXN* transcript levels compared with healthy controls (Chutake et al., 2014). Heterozygous carriers, who have one expanded *FXN* allele, have ∼50% transcript levels and remain asymptomatic. Thus, increasing the level of *FXN* transcript in FRDA patients to the level seen in heterozygous carriers is expected to be clinically beneficial. Approximately 20% of FRDA patients have one expanded GAA triplet-repeat that contains <500 triplets, and they tend to have more residual transcript (>15%), a later age of onset (≥15 years), slower progression, and longer lifespans (Durr et al., 1996; Montermini et al., 1997; Tsou et al., 2011). Thus, even a modest increase in *FXN* transcript may offer meaningful clinical benefit. *FXN* reactivation is an especially attractive therapeutic strategy in FRDA because the *FXN* coding sequence remains intact in the majority of patients, and the reactivated gene would likely benefit from the availability of endogenous regulatory elements.

The *FXN* gene is in heterochromatin form in FRDA, marked by hypoacetylation and trimethylation of specific histone lysine residues and co-incident DNA hypermethylation (reviewed by Yandim et al., 2013). Treatment with a class I histone deacetylase inhibitor (HDACi-109) reverses histone hypoacetylation, restores the abnormal *FXN* nucleosomal organization, and partially restores *FXN* transcript and frataxin protein in various pre-clinical FRDA models and in FRDA patients (Herman et al., 2006; Sandi et al., 2011; Soragni et al., 2014; Chutake et al., 2016). While HDAC inhibitor treatment is a promising strategy to reactivate the silenced *FXN* gene in FRDA, one of its limitations is the considerable inter-individual variability in drug response (Plasterer et al., 2013). The variable length of expanded repeats in FRDA does not explain this variability, leaving its molecular basis unknown.

A prominent epigenetic signature located in intron 1 of the *FXN* gene is an FRDA-specific differentially methylated region (FRDA-DMR), which shows >90% methylation in FRDA versus <10% in non-FRDA controls (Rodden et al., 2021). Bisulfite deep sequencing of the FRDA-DMR in patients show individual *FXN* strands that are variably methylated, providing a quantitative measure of epigenetic heterogeneity in somatic cells. Hypermethylation of the FRDA-DMR in FRDA, which is stable and reproducible, is broadly comprised of three types of somatic *FXN* epialleles: fully methylated, partially methylated, and unmethylated. The proportion of unmethylated epialleles accurately predicts *FXN* transcriptional deficiency and age of onset in patients (Rodden et al., 2021), suggesting that variably methylated *FXN* epialleles reflect different functional states of the *FXN* gene in somatic cells. Reasoning that fully methylated epialleles may represent completely silenced *FXN* genes that are relatively resistant to reactivation, we hypothesized that partially methylated and unmethylated epialleles likely represent potential targets for gene reactivation. If so, the variable prevalence of such epialleles may explain the inter-individual variability in response to HDACi-109. It should be noted that HDAC inhibitors are not designed to act directly on DNA methylation. Our model of gene silencing/reactivation assumes that the different *FXN* epialleles serve as surrogates for somatic *FXN* genes that have been variably silenced by the expanded GAA triplet-repeat in *cis*, and thus may have variable potential for gene reactivation. This is generally consistent with variegated epigenetic silencing, a mechanism of gene silencing attributed to expanded GAA triplet-repeats (Saveliev et al., 2003).

A prospective cohort of 50 FRDA patients was assessed for increase in *FXN* transcript via HDACi-109 treatment of PBMCs, as well as the prevalence of various *FXN* epialleles typed by bisulfite deep sequencing. The prevalence of unmethylated and partially methylated somatic *FXN* epialleles (0–64% methylation) correlated significantly with response to HDACi-109, and the prevalence of heavily methylated epialleles (>70% methylation) did not. This indicates that somatic epigenetic heterogeneity in FRDA explains, at least in part, the inter-individual variability in *FXN* reactivation via HDAC inhibition.

## Materials and Methods

### Study Participants and HDACi-109 Treatment

Peripheral blood samples were collected in purple-top/EDTA tubes from 50 study subjects with a confirmed DNA diagnosis of FRDA (homozygosity for expanded GAA triplet-repeat alleles; Table 1), 14 unaffected individuals, and five heterozygous carriers at The Children’s Hospital of Philadelphia (CHOP). Blood samples were shipped overnight on ice via courier service for processing and analysis at The University of Oklahoma Health Sciences Center (OUHSC) in Oklahoma City. Research Protocols were approved by the Institutional Review Boards at both institutions (CHOP IRB# 01-002609 and OUHSC IRB# 8071). Informed consent was obtained from all participants and/or their legal guardian(s) in accordance with the Declaration of Helsinki. PBMCs were isolated using Ficoll-Paque PLUS (GE Healthcare) and incubated in lymphocyte growth medium (RPMI with Glutamax, 15% FBS, pen/strep) at 37°C in 5% CO_2_ for 24 h. Cells were treated with 10 μM HDACi-109 (RG2833, Selleckchem, #S7292) dissolved in dimethyl sulfoxide (DMSO) or with DMSO only for 48 h. The final concentration of DMSO in the cell culture medium for HDACi-109 or DMSO-only treatment was 0.1%.

**TABLE 1:** Patient demographics.

Characteristic	Median (range)
Age of onset^#^, year	12.5 (4–41)
Disease duration^#^, year	16 (3–44)
GAA1*, triplets	633 (126–1025)
GAA2*, triplets	910 (280–1400)

#n = 48, *n = 49.

### RT-qPCR and Calculation of HDACi-109 Response

Total RNA (400 ng) was reverse transcribed following manufacturer instructions for the QuantiTect^®^ reverse transcription kit (Qiagen). Transcript levels were quantified by real-time PCR with Power SYBR green PCR master mix (Applied Biosystems) on a Roche LightCycler^®^ 96 System. Primers spanned the splice junction of *FXN* exons 3 and 4 values were normalized to expression of *RPL27* using the ΔΔCt method. Primer sequences and reaction conditions for *FXN* Ex3-Ex4 and *RPL27* were as previously described (Herman et al., 2006; de Jonge et al., 2007).

**FIGURE 1:**
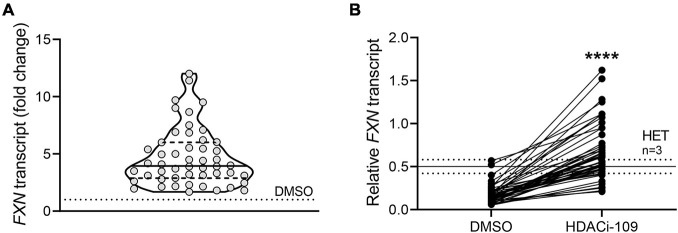
*FXN* gene reactivation with HDACi-109 in FRDA shows considerable inter-individual variability. PBMCs from 50 FRDA patients were treated with HDACi-109 or DMSO for 48 h. Response to HDACi-109 was calculated utilizing RT-qPCR measurements of *FXN* transcript. **(A)** Violin plot showing the distribution of fold-change in *FXN* transcript in PBMCs treated with HDACi-109, relative to *FXN* transcript in DMSO-control treated PBMCs for each of 50 FRDA patients. **(B)** *FXN* transcript in DMSO and HDACi-109 treated PBMCs was calculated relative to the *FXN* transcript level seen in heterozygous carriers (HET), which was artificially set to 0.5 (solid line labeled “HET” at *y* = 0.5 is the median level, dotted lines indicate standard deviation among 3 heterozygous carriers). ****p < 0.0001 (Mann–Whitney test).

**FIGURE 2:**
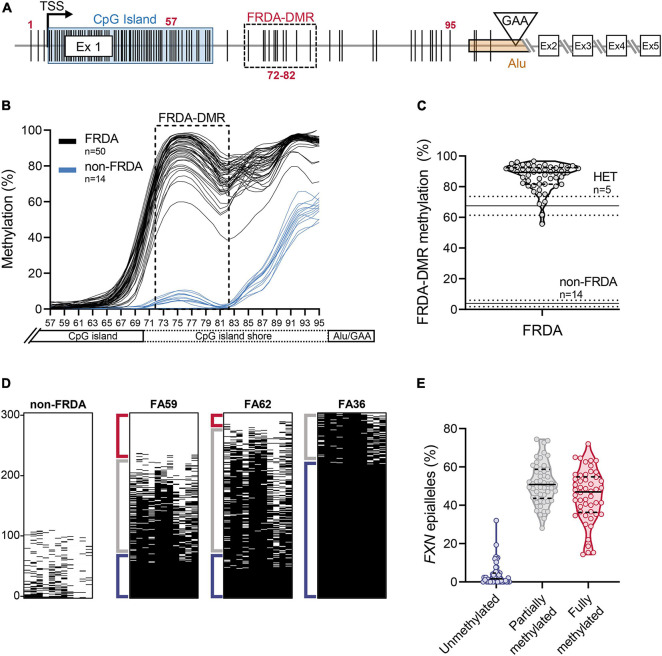
Heterogeneity in somatic *FXN* DNA hypermethylation in FRDA. **(A)** Schematic of the *FXN* gene with five coding exons (Ex 1–5), is shown focusing mainly on the proximal part of the *FXN* gene [adapted from Rodden et al. (2021)]. The schematic depicts the locations of individual CpG dinucleotides (vertical black lines, numbered 1–95; see section “Materials and Methods”), the transcriptional start site (TSS), *FXN* CpG island (blue box), and the GAA triplet-repeat in intron 1 [inverted triangle, within an Alu element (orange box)]. The FRDA-DMR is indicated by a black dashed box, which contains eleven CpG sites, numbered 72–82. In this study, CpG sites 57–95 (*n* = 39; that span the distance between the 3′ end of the CpG island and the GAA triplet-repeat) were typed for DNA methylation using bisulfite deep sequencing. **(B)** DNA methylation (%), calculated from *n* = 1000 sequence reads at each of the 39 CpG dinucleotides, numbered 57–95 on *X*-axis, is shown as LOWESS regression trend lines (Cleveland, 1981), for 50 FRDA patient-derived PBMCs (black lines) and 14 non-FRDA control PBMCs (blue lines). The FRDA-DMR, spanning CpG sites 72–82, is indicated by the dashed box. **(C)** DNA hypermethylation of the FRDA-DMR, as a percentage of *n* = 1000 reads, is plotted for *n* = 50 patients. Within the violin plot, the median (solid line) and demarcation of quartiles (dotted lines) are indicated. Horizontal lines across the graph: solid lines indicate median, dotted lines indicate standard deviation for *n* = 5 heterozygous carriers “HET” and *n* = 14 non-FRDA controls. **(D)** Bisulfite deep sequencing reads (*n* = 300) of the FRDA-DMR as a single amplicon, such that methylation status of all 11 CpGs are typed in *cis*, are vertically stacked, sorted with highest methylation at the bottom (black dash = methylated CpG). Representative results are shown for a single non-FRDA control, and three FRDA patients with variable proportions of unmethylated (≤2 CpGs methylated out of 11; Red bracket), partially methylated (>2 and <11 CpGs methylated out of 11; Gray bracket), and fully methylated (all 11 CpGs methylated; Blue bracket) somatic *FXN* epialleles. **(E)** The distribution of somatic *FXN* epiallele proportions in the FRDA-DMR of FRDA patient-derived PBMCs. Within the violin plots, the median (solid line) and demarcation of quartiles (dotted lines) are indicated.

Response to HDACi-109 was calculated in two ways: (1) *individual fold change* was calculated as the *FXN* transcript level for HDACi-109 treated PBMCs calculated relative to that individual’s DMSO-only level, which was artificially set to 1 (see Figure 1A); (2) *individual reactivation response relative to heterozygous carrier* was calculated using the *FXN* transcript level in DMSO-only and HDACi-109 treated PBMCs for all patients, relative to the *FXN* transcript level in heterozygous carriers, artificially set to 0.5 (see Figure 1B). The individual reactivation response relative to heterozygous carrier was used in all correlation analyses in this study.

### DNA Methylation Analysis

Bisulfite deep sequencing to assay DNA methylation at the *FXN* locus was previously described in detail (Rodden et al., 2021). Briefly, genomic DNA (0.5 μg), isolated from freshly isolated PBMCs, was bisulfite converted and prepared for targeted deep sequencing. Four amplicons were analyzed to cover all the CpG dinucleotides from the 3′ end of the CpG island to the expanded GAA triplet-repeat (*n* = 39 CpG sites; numbered 57–95 in Figure 2A), i.e., the region of the *FXN* gene known to be hypermethylated in FRDA. Amplicons were dual-indexed and pooled to create a library which was sequenced on an Illumina MiniSeq platform. *n* = 1000 sequence reads per sample were used to calculate the percentage of methylated cytosines at individual CpG dinucleotides and plotted with LOWESS regression to generate trend lines (Cleveland, 1981). Methylation in the FRDA-DMR, a region previously identified as having the maximal difference in methylation between FRDA (typically >90%) and non-FRDA controls (typically <10%), was calculated using *n* = 1000 sequencing reads covering the 11 CpG sites that map within the FRDA-DMR (numbered 72–82 in Figure 2A). Deep sequencing of a single amplicon that spanned all 11 CpG dinucleotides in the FRDA-DMR and analyzing the methylation status in *cis* within 300 individual reads (representing 300 *FXN* molecules per patient) was used to identify individual *FXN* epialleles. Epialleles were defined as follows: fully methylated (all 11 CpG sites methylated), unmethylated (≤2 CpG sites methylated), and partially methylated (>2 and <11 CpG sites methylated). The definition of partially methylated alleles was further refined in the analysis in Figure 4. Unmethylated epialleles were permitted to have up to 2 methylated CpGs because of the occasional and sporadic methylation seen in *FXN* epialleles from non-FRDA controls. Epiallele proportions were calculated as the percentage of *n* = 300 individually sequenced *FXN* DNA strands.

**FIGURE 3:**
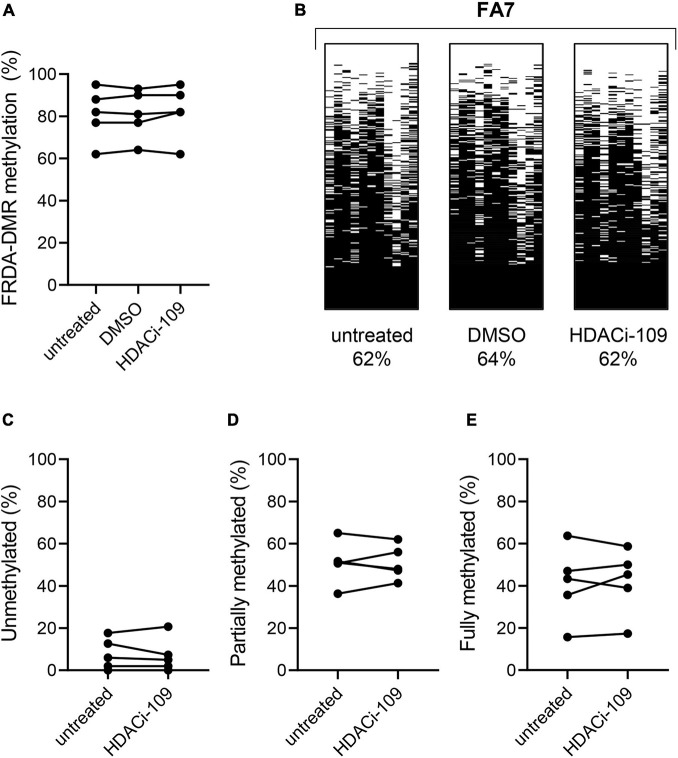
HDACi-109 treatment does not alter FRDA-DMR methylation. **(A)** Methylation levels (total % methylation in 300 sequence reads) in the FRDA-DMR of five FRDA patients are shown for the indicated three conditions: untreated, DMSO-control treated, and HDACi-109 treated PBMCs. **(B)** A representative patient is shown with the same three conditions (untreated, DMSO-control treated, and HDACi-109 treated), with labels indicating the total FRDA-DMR methylation (%) for each condition. Proportions (% of 300 reads) of **(C)** unmethylated, **(D)** partially methylated, and **(E)** fully methylated somatic *FXN* epialleles in the FRDA-DMR of the five FRDA patients, either untreated or treated with HDACi-109.

**FIGURE 4:**
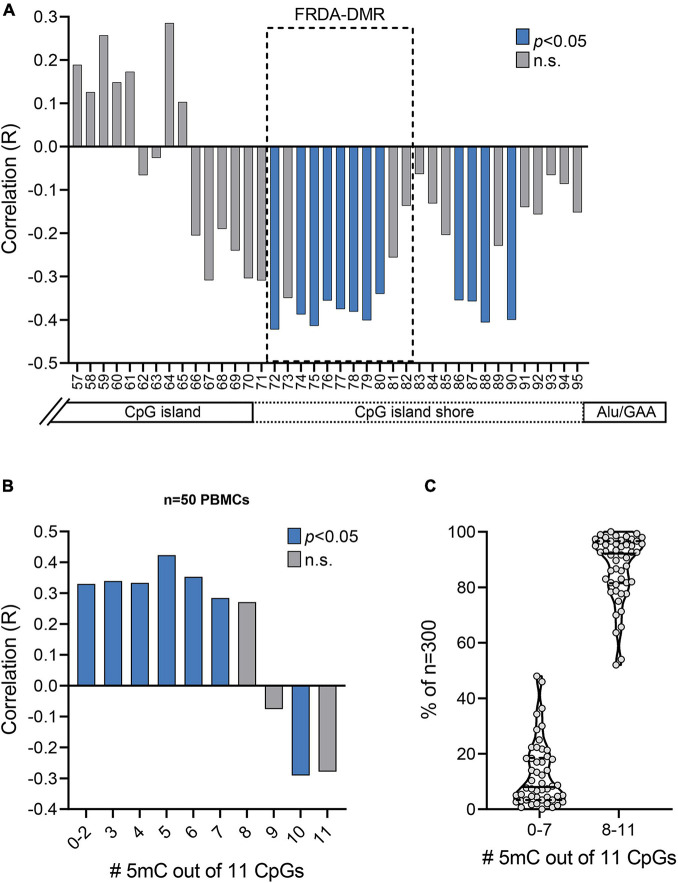
Prevalence of unmethylated and partially methylated *FXN* epialleles correlates with variable inter-individual response to HDACi-109 treatment in FRDA. **(A)** Correlation plot displaying *R* values for coefficient of correlations of HDACi-109 response with methylation (%) at each of the 39 individual CpG sites numbered 57–95 (as in Figure 2A). The FRDA-DMR, which contains 11 CpG sites numbered 72–82, is outlined with a dashed box. Blue bars indicate statistically significant correlations (*p* < 0.05; see Table 3) and gray bars represent correlations that are not significant (n.s.). **(B)** Correlation plot displaying *R* values for coefficients of correlation of HDACi-109 response with the prevalence of variably methylated *FXN* epialleles (either methylated at 0–2, 3, 4, 5, 6, 7, 8, 9, 10, or 11 of the 11 total CpG sites) in the FRDA-DMR. Blue bars indicate statistically significant correlations (*p* < 0.05; see Table 4) and gray bars represent correlations that are not significant (n.s.). 5mC = methylated cytosine. **(C)** Proportion of somatic *FXN* epialleles with 0–7 or 8–11 methylated CpG sites (out of 11 total CpGs) in the FRDA-DMR of *n* = 50 FRDA patients. Within the violin plots, the median (solid line) and demarcation of quartiles (dotted lines) are indicated. 5mC = methylated cytosine.

### Statistical Analyses

Statistical tests were performed using GraphPad, Prism v9. Student’s *t*-test and Mann–Whitney test were used to compare means and medians where appropriate. Linear correlations were evaluated to determine the correlation coefficient “R” where appropriate, and the method of Benjamini and Hochberg (1995) for false discovery rate (FDR = 10%) was used to account for multiple linear regression comparisons for the data in Figure 4A, and depicted as the *adjusted p* values in Table 3 (which had a modified discovery threshold of *p* < 0.04).

**TABLE 2:** Demographics of patients analyzed for the effect of HDACi-109 on DNA methylation.

Characteristic	Patient ID				
	FA1	FA3	FA4	FA6	FA7
Sex	M	F	M	F	M
Age of onset, year	4	15	17	11	15
GAA1, triplets	766	500	216	547	400
GAA2, triplets	1000	933	1149	993	700
FRDA-DMR methylation (%)	95	77	82	90	62
Unmethylated epialleles (% of 300)	0	13	6	2	19
Partially methylated epialleles (% of 300)	37	52	51	51	65
Fully methylated epialleles (% of 300)	63	35	43	47	15
HDACi-109 response (relative *FXN*)	0.53	0.87	1.01	1.11	1.25

**TABLE 3:** Correlation of methylation at individual CpG sites vs. HDACi-109 response in FRDA.

CpG dinucleotide ID	*p* value	Adjusted *p*^#^
72	0.002	0.031
74	0.005	0.035
75	0.003	0.031
76	0.011	0.041
77	0.007	0.036
78	0.006	0.035
79	0.004	0.031
80	0.012	0.041

86	0.012	0.041
87	0.011	0.041
88	0.004	0.031
90	0.004	0.031

*#Adjusted p values (Benjamini and Hochberg, 1995), see section “Materials and Methods.”*

*CpG dinucleotides above the solid line are within the FRDA-DMR.*

## Results

### *FXN* Gene Reactivation in Friedreich Ataxia via HDACi-109 Shows Considerable Inter-Individual Variability

A prospective cohort of 50 FRDA patients was enrolled with the goal of testing the hypothesis that *FXN* DNA hypermethylation predicts response of *FXN* gene reactivation via HDACi-109 treatment. This cohort was generally representative of the broader FRDA patient population in terms of age of onset and sizes of expanded GAA triplet-repeats (Table 1; Regner et al., 2012). Consistent with previous studies, HDACi-109 treatment of PBMCs increased the median steady-state *FXN* transcript level to 3.9-fold over the corresponding DMSO control (Figure 1A), indicating that it reactivates the silenced *FXN* gene in FRDA. However, individual responses varied considerably in the increase in *FXN* transcript following HDACi-109 treatment, ranging from 1.7- to 12-fold over DMSO control (Figure 1A). A desirable goal of *FXN* reactivation therapy is to increase *FXN* transcript levels to at least the level seen in asymptomatic carriers, i.e., to approximately 50% of non-FRDA levels. In FRDA there is considerable variability in the residual level of *FXN* transcript, mostly driven by the large variability in length of the expanded GAA repeats, especially the shorter of the two expanded alleles (GAA1) (Pianese et al., 2004; Chutake et al., 2014; Li et al., 2015). Thus “fold-change over DMSO control” as a measure of drug response (as seen in Figure 1A) lacks the ability to demonstrate the potential clinical usefulness of the magnitude of increase in *FXN* transcript level. Therefore, the increase in *FXN* levels with HDACi-109 was analyzed relative to the levels seen in heterozygous carriers, which was artificially set at 0.5 to represent half the level seen in non-FRDA controls (Figure 1B). Using this measure, the cohort showed an increase in *FXN* transcript level from a median value of 0.15 (range: 0.05–0.57) to 0.59 (range: 0.21–1.62) following HDACi-109 treatment (*p* < 0.0001; Figure 1B), and resulted in samples from 42 of 50 patients (84%) achieving or surpassing the steady-state levels of *FXN* transcript seen in heterozygous carriers. Thus, HDACi-109 effectively reactivates the silenced *FXN* gene in FRDA, but with considerable inter-individual variability, whereby a substantial minority of individuals fail to achieve a level that is considered clinically meaningful.

### Somatic Heterogeneity of *FXN* DNA Hypermethylation in Friedreich Ataxia

In order to test the hypothesis that inter-individual variability in *FXN* DNA methylation predicts variability in HDACi-109 response, *FXN* DNA methylation was analyzed in PBMCs from the cohort of 50 patients. The expanded GAA triplet-repeat in FRDA induces DNA hypermethylation in intron 1 (Castaldo et al., 2008; Evans-Galea et al., 2012; Rodden et al., 2021). The maximal difference of DNA methylation between FRDA and non-FRDA controls is seen in a region of intron 1 with 11 contiguous CpG sites, which form a defined FRDA-specific differentially methylated region (FRDA-DMR) located just downstream from the *FXN* CpG island (Figure 2A; Rodden et al., 2021). This cohort showed the expected variation in magnitude of FRDA-DMR methylation, ranging from 56 to 97% in FRDA (*n* = 50; Figures 2B,C; Supplementary Table 1), compared with 4% in non-FRDA controls (*n* = 14; *p* < 0.0001; Figures 2B,C) and 68% in heterozygous carriers (*n* = 5; *p* < 0.001; Figure 2C). Deep sequencing of a single amplicon spanning the FRDA-DMR to assess methylation of all 11 CpG sites in *cis* was performed to analyze methylation within individual *FXN* DNA strands, i.e., somatic *FXN* epialleles. DNA methylation of 300 such *FXN* epialleles, representing the somatic variability of methylation in the FRDA-DMR, was determined for all 50 patients in our cohort (Figure 2D shows a representative set of 300 stacked *FXN* strands per individual; black dash = methylation; sorted with highest methylation at the bottom). Individually sequenced *FXN* molecules revealed three types of epialleles: fully methylated (with all 11 CpGs methylated), unmethylated (≤2 CpGs methylated), and partially methylated (with >2 and <11 CpGs methylated) (Figures 2D,E). The majority of epialleles in FRDA patients are fully methylated or partially methylated, both displaying considerable inter-individual variability in our cohort (Figures 2D,E). Patients also showed a smaller and variable proportion of unmethylated epialleles (Figure 2E). We hypothesized that the highly variable prevalence of *FXN* epialleles may help explain the inter-individual variability in response to HDACi-109 treatment. Specifically, that the relative prevalence of partially methylated and unmethylated epialleles in FRDA may represent the individual potential for *FXN* reactivation.

### HDACi-109 Treatment Does Not Alter FRDA-Specific Differentially Methylated Region Methylation

*FXN* DNA hypermethylation in FRDA is known to be primarily dependent on the length of the expanded GAA repeat, and does not correlate with patient age and disease duration, or differ between males and females (Rodden et al., 2021). Our hypothesis that variability in HDACi-109 response is predicted by variability in *FXN* DNA methylation requires that FRDA-DMR methylation and the distribution of somatic *FXN* epialleles remain stable. This requires that drug treatment *per se* does not alter the level of DNA methylation or relative proportion of *FXN* epialleles. Five FRDA patients in our cohort were systematically tested to investigate if HDACi-109 alters the level of FRDA-DMR hypermethylation, or the proportional distribution of somatic *FXN* epialleles. These individuals were selected for their wide range of GAA repeat lengths, DNA methylation levels, and broad distribution of the three types of *FXN* epialleles (Table 2). These five patients also showed robust reactivation responses following HDACi-109 treatment, achieving at least the level seen in heterozygous carriers (set at 0.5; Table 2), indicating adequate modification of the *FXN* locus. DNA methylation was assayed with and without drug treatment, which did not appreciably change in these patients upon treatment with HDACi-109 (Figure 3A). We also did not observe any appreciable change in the prevalence of unmethylated, partially methylated, and fully methylated epialleles in these patients following treatment (Figures 3B–E). Therefore, at least in non-dividing PBMCs, HDACi-109 treatment does not alter the magnitude or pattern of FRDA-DMR hypermethylation in FRDA.

### Prevalence of Unmethylated and Partially Methylated *FXN* Epialleles Correlates With Variable Inter-Individual Response to HDACi-109 Treatment in Friedreich Ataxia

Bisulfite deep sequencing provided a rich dataset of DNA hypermethylation in FRDA, including 300 *FXN* epialleles representing the somatic heterogeneity of FRDA-DMR hypermethylation in each of 50 patients. The level of DNA methylation at each of the 39 CpG sites (numbered as 57–95 in Figure 2A) spanning the region of intron 1 upstream of the expanded GAA repeat was tested for correlation with response to HDACi-109. This revealed that most CpG sites within the FRDA-DMR (and a few outside of the DMR) showed statistically significant negative correlations with HDACi-109 response (Figure 4A and Table 3), indicating that lower methylation in the FRDA-DMR correlates with a higher magnitude of response. We then tested for correlation between the prevalence of variably methylated *FXN* epialleles and response to treatment with HDACi-109. HDACi-109 response showed a statistically significant positive correlation with the prevalence of unmethylated (≤2 CpGs methylated) epialleles and partially methylated epialleles containing 3–7 (of 11) methylated CpG sites (Figure 4B and Table 4). Interestingly, methylated epialleles with ≥8 CpGs methylated and fully methylated *FXN* epialleles did not show this correlation, or were even inversely correlated (Figure 4B and Table 4), suggesting that highly methylated epialleles (>70%) represent *FXN* genes that are relatively resistant to reactivation. These data support the hypothesis that the variable prevalence of unmethylated and partially methylated *FXN* epialleles (0–64% methylation) underlies the potential for *FXN* reactivation via HDACi-109 treatment in FRDA. Indeed, such alleles constitute a substantial, albeit variable, subset of somatic *FXN* genes in FRDA patients (Figure 4C).

**TABLE 4:** Correlation of variably methylated *FXN* epialleles vs. HDACi-109 response in FRDA.

Methylated CpGs out of 11 (No.)	*p* value^#^
0–2	**0.020**
3	**0.016**
4	**0.018**
5	**0.002**
6	**0.012**
7	**0.045**
8	0.057
9	0.609
10	**0.041**
11	0.050

*#p values in bold typeface are statistically significant.*

Previous studies did not find any correlation of GAA1 with response to HDACi-109 treatment (Plasterer et al., 2013). In our cohort we noted weak correlation of response to HDACi-109 with GAA1 (*p* = 0.04). No correlation was seen with GAA2 (*p* = 0.62) or GAA1 + 2 (*p* = 0.12). Furthermore, no correlation of HDACi-109 response was seen with either disease duration (*p* = 0.12) or patient age at sampling (*p* = 0.74).

## Discussion

Deep sequencing of individual *FXN* molecules, wherein all the 11 CpG sites spanning the FRDA-DMR are assayed in *cis* for their methylation status, revealed the remarkable level of heterogeneity of variably methylated *FXN* epialleles borne by somatic cells in FRDA. This rich dataset permitted a nuanced exploration of the relationship between DNA hypermethylation in FRDA and its potential role in *FXN* gene reactivation. Our data support a model of gene silencing in FRDA in which the subset of unmethylated or partially methylated *FXN* epialleles (with <70% methylation) represent a pool of somatic *FXN* genes that are functionally different from the heavily methylated epialleles (with >70% methylation). The data suggest that the subset of *FXN* epialleles with <70% methylation underlies the potential for *FXN* reactivation via HDACi-109 treatment in FRDA, and such alleles constitute a substantial subset of somatic *FXN* genes in FRDA patients. Conversely, our data suggest that *FXN* epialleles with >70% methylation represents a pool that is relatively resistant to reactivation. It should be noted that *FXN* methylation, being used here as a highly quantitative readout, is likely serving as a surrogate for some underlying “correctable” epigenetic signature that allows for reactivation via HDAC inhibition. Nevertheless, our data indicate, for the first time, that somatic heterogeneity of epigenetic silencing signals in FRDA plays a role in determining inter-individual variability in *FXN* gene reactivation. The prevalence of unmethylated epialleles in the FRDA-DMR have previously been shown to play a role in determining *FXN* transcript levels and age of onset in FRDA, explaining ∼50% of the variability (Rodden et al., 2021). Altogether, these observations support an epigenetic silencing mechanism induced by the expanded GAA triplet-repeat in FRDA that is akin to variegated silencing (Saveliev et al., 2003), i.e., somatic mosaicism for functionally silenced, partially silenced yet amenable to reactivation, and non-silenced *FXN* epialleles.

A potential caveat of our analysis is that at least some of the individual *FXN* epialleles we typed may represent reamplification of the same *FXN* gene. However, this is unlikely to be a major confounder because of the reproducibility in methylation level and epiallele distribution noted upon resequencing the same PBMC sample, as seen in Figure 3. Similarly, we previously estimated the variability in FRDA-DMR methylation to be <3% S.D. when resequencing the same sample, over a range of methylation levels; and <1.5% S.D. when using a range of starting DNA concentrations of the same sample (Rodden et al., 2021). This reproducibility indicates that the sequencing read depth of 300 is likely capturing a representative sample of somatic *FXN* epialleles, although we cannot rule out some reamplification. Another caveat is that expanded GAA triplet-repeats are somatically unstable and that the *FXN* molecules with shorter repeats are the *FXN* epialleles with low levels of methylation. It is reasonable to expect that this would play some role, however, we have previously demonstrated (Sharma et al., 2002) and also argued (Rodden et al., 2021) that this role is likely to be limited. Essentially, the frequency of somatic reversion of the expanded GAA triplet-repeat in PBMCs (0.29%) is considerably lower than the observed prevalence of unmethylated epialleles in FRDA. Furthermore, the frequency of large contractions is higher in individuals with long GAA triplet-repeats and it is in these individuals we encounter the lowest prevalence of unmethylated epialleles.

The coefficients of correlation for the subtypes of variably methylated epialleles are moderate, suggesting that not all *FXN* molecules with <70% methylation are amenable to reactivation. This is a reasonable expectation because we wouldn’t expect congruency of all epigenetic silencing signals in every single *FXN* molecule, perhaps especially in those which are variably methylated. The data suggest that a subset of these molecules is amenable to reversal of hypoacetylation and restoration of the nucleosomal architecture. However, the observation that most CpG sites in the FRDA-DMR correlate individually with gene reactivation, and these same CpG sites were previously found to contribute to the severity of *FXN* gene silencing, supports a role for the FRDA-DMR in regulating expression of the *FXN* locus. Carefully designed single-cell assays will be needed to characterize the true nature of epigenotypic heterogeneity in FRDA. The ability to predict *FXN* reactivation in individual patients is a worthwhile endeavor that could help in optimizing its applicability as a therapeutic strategy in FRDA.

## Data Availability Statement

The original contributions presented in the study are included in the article/Supplementary Material, further inquiries can be directed to the corresponding author.

## Ethics Statement

Research Protocols were approved by the Institutional Review Boards at: The Children’s Hospital of Philadelphia (IRB# 01-002609) and The University of Oklahoma Health Sciences Center (IRB# 8071). Written informed consent to participate in this study was provided by the participant or participants’ legal guardian/next of kin.

## Author Contributions

LR, KG, and CL performed the experiments. SB was involved in planning and supervision of the project and procured funding for the project. LR, KG, CL, and SB processed and analyzed the data. LR and SB drafted the manuscript and designed the figures. DL aided in interpreting the results, worked on the manuscript, and provided key patient-derived samples along with clinical information. All authors discussed the results and commented on the manuscript.

## Conflict of Interest

The authors declare that the research was conducted in the absence of any commercial or financial relationships that could be construed as a potential conflict of interest.

## Publisher’s Note

All claims expressed in this article are solely those of the authors and do not necessarily represent those of their affiliated organizations, or those of the publisher, the editors and the reviewers. Any product that may be evaluated in this article, or claim that may be made by its manufacturer, is not guaranteed or endorsed by the publisher.
