# Supplementary material for: Epigenetic Heterogeneity in Friedreich Ataxia Underlies Variable FXN Reactivation
Source: Front Neurosci. 2021 Nov 25;15:752921. doi: 10.3389/fnins.2021.752921 (PMC8655727; doi:10.3389/fnins.2021.752921)
Supplement: Supplementary file 1 [file Table_1.pdf]

Subject ID	FRDA-DMR Methylation (%)
**FA1**	95
**FA3**	76.7
**FA4**	81.7
**FA5**	87.5
**FA6**	89.7
**FA7**	61.6
**FA8**	80.5
**FA10**	83.8
**FA11**	89.1
**FA12**	91.9
**FA13**	91.8
**FA14**	81.5
**FA15**	89
**FA16**	85.9
**FA17**	92.8
**FA20**	75.1
**FA21**	92.2
**FA22**	80.3
**FA23**	93.9
**FA24**	90
**FA25**	94.4
**FA26**	89.5
**FA27**	86.1
**FA28**	91.4
**FA29**	91.8
**FA30**	91.1

Subject ID	FRDA-DMR Methylation (%)
**FA31**	80.8
**FA32**	82.3
**FA33**	77.9
**FA34**	93.1
**FA35**	92.8
**FA36**	96.6
**FA37**	96
**FA43**	94.5
**FA44**	91.2
**FA47**	93.6
**FA48**	94.4
**FA50**	90.1
**FA51**	86.4
**FA52**	85.2
**FA53**	92.2
**FA54**	89.7
**FA55**	85.3
**FA56**	81.7
**FA57**	93.5
**FA58**	87.7
**FA59**	55.6
**FA60**	68.7
**FA61**	74.8
**FA62**	70.2
